# Longitudinal comparative evaluation of the equivalence of an integrated peer-support and clinical staffing model for residential mental health rehabilitation: a mixed methods protocol incorporating multiple stakeholder perspectives

**DOI:** 10.1186/s12888-016-0882-x

**Published:** 2016-06-02

**Authors:** Stephen Parker, Frances Dark, Ellie Newman, Nicole Korman, Carla Meurk, Dan Siskind, Meredith Harris

**Affiliations:** Metro South Addiction and Mental Health Service, 128 Main Street, Redland Bay, QLD 4162 Australia; The University of Queensland, Herston, Australia; University of Queensland School of Medicine, Herston, Australia

**Keywords:** Protocol, Mixed methods, Qualitative methods, Grounded theory, Rehabilitation, Peer support, Consumer involvement, Community care unit, Schizophrenia

## Abstract

**Background:**

A novel staffing model integrating peer support workers and clinical staff within a unified team is being trialled at community based residential rehabilitation units in Australia. A mixed-methods protocol for the longitudinal evaluation of the outcomes, expectations and experiences of care by consumers and staff under this staffing model in two units will be compared to one unit operating a traditional clinical staffing. The study is unique with regards to the context, the longitudinal approach and consideration of multiple stakeholder perspectives.

**Methods/design:**

The longitudinal mixed methods design integrates a quantitative evaluation of the outcomes of care for consumers at three residential rehabilitation units with an applied qualitative research methodology. The quantitative component utilizes a prospective cohort design to explore whether equivalent outcomes are achieved through engagement at residential rehabilitation units operating integrated and clinical staffing models. Comparative data will be available from the time of admission, discharge and 12-month period post-discharge from the units. Additionally, retrospective data for the 12-month period prior to admission will be utilized to consider changes in functioning pre and post engagement with residential rehabilitation care. The primary outcome will be change in psychosocial functioning, assessed using the total score on the Health of the Nation Outcome Scales (HoNOS). Planned secondary outcomes will include changes in symptomatology, disability, recovery orientation, carer quality of life, emergency department presentations, psychiatric inpatient bed days, and psychological distress and wellbeing. Planned analyses will include: cohort description; hierarchical linear regression modelling of the predictors of change in HoNOS following CCU care; and descriptive comparisons of the costs associated with the two staffing models. The qualitative component utilizes a pragmatic approach to grounded theory, with collection of data from consumers and staff at multiple time points exploring their expectations, experiences and reflections on the care provided by these services.

**Discussion:**

It is expected that the new knowledge gained through this study will guide the adaptation of these and similar services. For example, if differential outcomes are achieved for consumers under the integrated and clinical staffing models this may inform staffing guidelines.

## Background

Community based residential rehabilitation for mental health consumers in Australia has become increasingly available through non-government organisations (NGOs) and public health services [1]. These are bed-based services that focus on improving the independence and community functioning of persons affected by severe and persisting mental illness, predominantly those with a diagnosis of schizophrenia. The growth in availability of residential rehabilitation over the past 20 years has been linked in part to the recovery movement and research evidence promoting a more optimistic view of the potential for improvement among people with severe mental illness than has been previously assumed [2–4]. This paradigm shift has impacted the landscape of mental health policy and practice [5], facilitating a more holistic approach to treatment planning, and increasing the focus on addressing consumers’ functioning and attainment of personal goals [6]. However, at this time, there is limited evidence to guide service users, service providers and funding bodies about the effectiveness of residential rehabilitation service models [2] and how they should function.

There is limited research examining the outcomes of clinically focused community residential mental health rehabilitation services in Australia; much of what is available focuses on the consumers following their deinstitutionalisation [7]. With regards to non-clinical services, a 2012 consultation paper commissioned by the state of Victoria recommended discontinuation of bed-based adult rehabilitation services due to a lack of evidence of consumer outcomes and recovery oriented care [8]. Despite limitations in the evidence base, there has been substantial recent investment in additional capacity by the Queensland Government, with six new clinically operated community residential mental health rehabilitation services (126 beds) expected to open over the 2015–2016 period [9]. Novel approaches to the inclusion of peer workers have been considered for these units. There is a paucity of research to guide policy-makers, service providers and users as to the differences between models of staffing of residential mental health rehabilitation with regards to consumer preferences and outcomes. Better evidence about the effectiveness of these services, as well as the implications of integrating peer support workers into staffing models, is clearly needed.

Another important aspect of mental health policy and practice linked to the recovery movement is the increasing emphasis on the availability of peer support [10–12]. The concept of peer support has been formalised in roles such as ‘peer workers’ or ‘peer support workers’ where an individual with a lived experience of mental illness is employed with the expectation these experiences will be explicitly utilised in supporting consumers of the service [13]. It is argued that this lived experience facilitates the sharing of experiential knowledge about coping strategies and pathways to recovery. Peer support roles are being increasingly encouraged and trialled as a component of mental health service provision. However, despite this strong policy backing, available evidence regarding the effectiveness of peer support has been criticised with regards to methodological rigor and potential bias [10, 13]. Much of the evidence relating to the value of peer support is qualitative in nature, and quantitative studies have been completed in outpatient and inpatient settings rather than community residential services. Notwithstanding these limitations, the literature suggests that peer workers employed as case managers achieve equivalent outcomes for service users in comparison with professional case managers, with potential advantages in terms of consumer engagement and benefits for peer workers and the services they work with [12–15]. The possibility of improved engagement through the inclusion of peer-support is promising, particularly given increasing interest in the impact of consumer engagement on rehabilitation outcomes [16, 17].

Different ways of incorporating peer workers into rehabilitation have been considered by non-clinical NGO services and clinically focussed public mental health services. Some of these services have utilised ‘buy in’ approaches whereby clinical time or peer support is made available from an external provider. Additionally, clinical services have increasingly employed peer workers in positions that provide advocacy and support to facilitate improved understanding of consumer perspectives by the clinical team [18]. Each of these approaches have potential limitations. The employment of sole peer workers may be tokenistic, and there is a risk of peer worker values and attitudes assimilating with those of clinical staff over time. There may also be resistance to peer roles from clinical staff [13, 18]. Buy-in staffing models may not favour the potential for cross-fertilization of recovery paradigms that can occur from representing the peer work force as another discipline within a more integrated staffing model [19].

### Evaluation context

Continuing Care Units (CCUs) are a model of community-based residential rehabilitation care established in Australia in the 1990s as an alternative to long term psychiatric hospitalisation [20]. The model involves cluster housing in a community setting, with onsite mental health professionals providing individualised care with the purpose of maximising independence in the community. CCUs were initially intended to provide permanent accommodation and support, however, the philosophy of care has evolved. While the focus on delivering individualised care remains, the units no longer focus on providing lifelong care for formerly institutionalised consumers [7, 21]. Rather, they provide time-limited rehabilitation support, currently between 6 and 24 months, preparing individuals to live more independently in an alternative residential setting [22]. There is evidence to suggest that CCUs were able to meet their original purpose of providing an alternative and preferable care environment to long-term hospitalisation [7, 23]. However, many consumers continued to have significant disability and unmet needs post-discharge from the CCU [7]. Despite CCU availability since the 1990s there has been limited literature exploring their outcomes since their inception, or the experiences of care from the perspectives of staff and consumers [7, 24].

In 2012 the Metro South Addiction and Mental Health Service (MSAMHS) opened the first of three planned CCUs in the inner suburb of Coorparoo, in the city of Brisbane, Australia (see Table 1). Two more units have been opened in the outer-suburbs of Bayside and Logan in 2014/2015. All three services provide independent living units with the availability of 24-h staff support. The Coorparoo unit has a traditional clinical staffing model with a manager, occupational therapist, psychologist, social worker, nursing and medical staff (a ‘clinical staffing model’). The staffing at Bayside and Logan involves collaboration between clinical staff and peer workers (an ‘integrated staffing model’) [19]. Under the integrated model, peer workers replace non-senior nursing roles with the responsibility for implementing individualised rehabilitation plans for consumers. Peer workers comprise 61 % and 56 % of full-time equivalent staff at the Bayside and Logan CCU sites respectively. Unique features of the integrated model are the direct collaboration between peer workers and clinical staff within a single team, and peer support workers fulfilling the role of principal support or case manager in the day-to-day delivery of care.

**Table 1:** Characteristics of the Coorparoo, Bayside and Logan CCUs, and their referring districts

			Coorparoo CCU	Bayside CCU	Logan CCU
Location		Distance from state capital (km)	4.2	30.9	21.2
		LGA Index of Relative Socio-economic Disadvantage, 2011^a^ [85]	90	83	46
Referring district		Population [86]	588,475	143,628	287,517
		Availability of acute inpatient services	Yes	Yes	Yes
		Availability of community mental services	Yes	Yes	Yes
		Availability of inpatient rehabilitation mental health beds	No	Yes	No
		Availability of transitional housing team	Yes	No	No
		Availability of community based non-residential rehabilitation team	Yes	No	Yes
		Availability of dedicated mental health homelessness team	Yes	No	Yes
CCU	Philosophy of care	Recovery-oriented	Yes	Yes	Yes
		Strengths-based	Yes	Yes	Yes
		Designated rehabilitation focus	Yes	Yes	Yes
		Voluntary engagement in rehabilitation^b^	Yes	Yes	Yes
		Individualised care planning	Yes	Yes	Yes
		Transitional support	Yes	Yes	Yes
		Role of peer support in care planning and delivery	Limited	Focussed	Focussed
	Physical environment	Maximum occupancy (consumers)	20	20	16
		Number of self-contained independent living units	20	20	15
		Number of disabled access units	1/20	1/20	1/15
		Shared recreation and leisure facilities	Yes	Yes	Yes
	Available treatment & support	Individual psychotherapy support (CBT)	Yes	Yes	Yes
		Living skills support and development	Yes	Yes	Yes
		Structured leisure and physical activities	Yes	Yes	Yes
		Evidence based therapeutic group programmes	Yes	Yes	Yes
	Staffing	Staffing model	Clinical	Integrated	Integrated
		Total FTE staff	21.5	24.5	18.4
		Total FTE peer-support staff	0.6	16	10.4
		Total FTE clinical staff	19.9	7.5	7
		Peer support : Clinical staff ratio	0.03	2.13	1.49
		Staff : Consumer ratio	1.08	1.23	1.15

^a^Local Government Area (LGA) percentile rank of Relative Socio-economic Disadvantage in comparison to all other LGAs in Australia

^b^Involuntary consumers are accepted at all three CCUs with explicit emphasis on voluntary engagement in available rehabilitation activities

There is an expectation that similar care will be delivered across the CCUs operating the integrated and clinical staffing models. The processes for staff orientation and training at the Bayside and Logan sites were modelled from those initially implemented at the Coorparoo CCU. All three of the MSAMHS CCUs operate under a common state-wide model of service [22] and have a shared governance structure. The key distinction between these sites is their staffing models and geographic locations (see Table 1). The current iteration of the model of service emphasises provision of recovery oriented rehabilitation and support to consumers with severe and persistent mental illness to assist them towards personal recovery. Definitions of recovery and recovery-oriented practice in these services are taken from The National Framework for Recovery-oriented Mental Health Service (NFROMHS) [25]. This framework defines recovery oriented practice as that which:

1. “Recognises and embraces the possibilities for recovery and wellbeing created by the inherent strength and capacity of all people experiencing mental health issues
2. Maximises self-determination and self-management of mental health and wellbeing
3. Assists families to understand the challenges and opportunities arising from their family member’s experiences (p3).”

The concept of personal recovery is defined under the framework as: “being able to create and live a meaningful and contributing life in a community of choice with or without the presence of mental health issues” (p2).

The model of service provides the following explanation of who CCU services are for:

“The CCU service is for adult consumers aged 18 to 65 years who have a severe and persistent mental illness. The symptoms substantially impair their psychosocial function and the capacity for independent living. These consumers have, or are likely to have, difficulty functioning within their community and have had problems accessing other services for support. It is expected that CCU consumers will benefit from 24-hour mental health care.

The diagnostic profile of eligible consumers includes individuals with a range of diagnoses associated with a severe and persistent mental illness, a cohort of whom may also have a comorbid drug or alcohol disorder. Consumers whose treatment is complicated by an intellectual disability and/or personality disorder are also eligible for this recovery-based consumer rehabilitation program. Consumers who benefit from participating are often not acutely unwell but in a recovery phase and readjusting to living in a social setting and require more support than is available to them in other settings such as home or social housing. This includes consumers who have symptoms that may be slow to respond to treatment or experience behavioural disturbances that make living in alternative community settings difficult” (p7).

The aforementioned gaps in the literature regarding the outcomes of engagement with CCU care, and experiences of care, are concerning given the investment in new service capacity that has occurred over recent years. The opening of the two new CCUs has presented a unique opportunity to evaluate the outcomes and experiences of care associated with CCUs offering an integrated staffing model in comparison to a traditional clinical staffing model. Understanding consumer perspectives is critical to ensuring service delivery contributes to recovery outcomes. Additionally, understanding staff expectations and experiences of working in these services presents an opportunity to explore the function of these units and the extent to which this aligns with the designated model of service. This study protocol outlines the proposed collection of evaluative data from both consumers and staff, across multiple stages of the implementation of the new staffing model. Data from the clinical staffing model will be collected to facilitate comparative analysis between traditional clinical and integrated staffing models.

### Aims and benefits

The study examines the outcomes of engagement with CCUs, and considers the impact of the integrated staffing model on both the outcomes and experiences of care in comparison to a clinically staffed CCU.

The quantitative component of the study explores the expectation that consumers under integrated and clinical staffing models will experience improvement in psychosocial functioning as measured by the total Health of the Nation Outcome Scale (HoNOS) [26] score following discharge from CCU care, and that no significant differences will emerge between the changes observed under each model. Equivalence testing rather than assessing for superiority or non-inferiority of the integrated staffing model is supported by:

1. The operation of an identical model of service across both the clinical and integrated sites, hence the expectation that equivalent care will be delivered. If equivalent care was realised was a staffing model where the majority of staff are employed on the basis of their ‘lived experience’ rather than clinical expertise this would support future decision-making about staffing on the basis of secondary considerations [27] such as cost-effectiveness and consumer preference.
2. Limitations in the quantitative evidence base relating to the impact of the integration of peer support to justify a hypothesis of superiority. Where quantitative findings are available these have tended to discuss equivalent outcomes to clinical staff. Furthermore, a failure to demonstrate superiority of the integrated model would not answer the question of whether such a staffing approach may be appropriate to be considered by other services [28].
3. The limitations of testing a non-inferiority hypothesis with regards to establishing the equivalence of the two models where qualitative differences in the experience of care are theorised, and the impact of these on consumer outcomes is unknown.

In addition to the primary outcome of focus, the quantitative data collection and analyses will consider the following questions:

1. Who are the consumers who currently utilise community based residential rehabilitation services (cohort description)?
2. Does provision of CCU rehabilitation care result in change in domains of relevance to serious and persistent mental illness between admission and discharge (secondary outcomes)?
3. What factors predict a consumer psychosocial outcome through the provision of CCU based rehabilitation support (hierarchical linear regression modeling)?
4. How do the operational costs associated with running a CCU under the clinical and integrated staffing models compare (description)?

The qualitative aspects of the study will facilitate detailed understanding the nature of the services under investigation. Following a pragmatic approach to grounded theory enquiry, no hypotheses are made a priori in relation to the qualitative data. Social and social-psychological phenomena underlying the consumer and staff experience of CCU based rehabilitation will be explored, including:

1. Defining and comparing the expectations of care at the CCU under the integrated staffing model for consumers and staff
2. Comparing the experience of care in a traditional clinical staffing model with the novel integrated model for both consumers and staff
3. Examining reflections on the experience of rehabilitation care post-discharge for consumers under both the clinical and integrated CCU staffing models.

Principles of participatory action research [29] have informed the approach to the qualitative component of the study. Both consumer and staff participants will be actively involved as co-creators of knowledge during the analysis and manuscript preparation. Additionally, this knowledge is expected to drive future service development.

The understanding of the residential rehabilitation service and the implications of the integrated staffing model achieved through this project will have implications for future service planning. The outcomes will be relevant to the staffing and processes of similar units in Australia, as well as guiding service providers internationally about novel ways of incorporating peer support into routine clinical care.

## Methods/design

The mixed methods approach will facilitate understanding of not only ‘what’ works but also consider ‘why’ this works and for whom, as well as ‘how’ similar services may be modified to better meet the needs of consumers. The approach has relevance given the socially complex nature of these services [30], the limited literature examining similar services internationally, and the acknowledged uncertainty as to exactly what mental health rehabilitation services are and whom they should be for [31]. Whilst the quantitative and qualitative aspects will be dissociable, the qualitative analysis will provide depth to understanding emergent outcomes as well as the opportunity to complete secondary analyses focussing on subgroups of participants achieving favourable and unfavourable outcomes through the provision of residential rehabilitation support.

## Ethical approval and informed consent

Both the University of Queensland and the Metro South Human Research Ethics Committee have provided ethical clearance for this study (HREC/14/QPAH/62). Participation of both staff and consumers is on the basis of voluntary informed consent. The CCUs accept consumers aged 18–65 who have a severe and persistent mental illness, predominantly schizophrenia, that substantially impairs psychosocial functioning and capacity for independent living [32]. For those consumers lacking capacity to provide this consent, the consent of their relevant guardian for the inclusion of routine assessment and outcomes data in the evaluation will be sought. The nature of the care delivered to a consumer at the CCUs will not be altered by study participation. A small gratuity for consumers involved in the semi-structured individual interviews is provided. This gratuity recognises the investment of participant time, and the limited direct benefits through participation.

### Quantitative methodology

#### Design

A longitudinal, prospective cohort design is used to examine outcomes of consumers under the clinical and integrated staffing models. The methodology has been developed with consideration of the STROBE Statement [33] and it is intended that results will be reported in a manner consistent with these guidelines. Principles of relevance to the quality reporting of equivalence studies have also been considered [34]. Prospective data will be obtained from a clinical assessment battery at three time points: admission, discharge and the 0–12 months post-discharge (see Table 2). Supplementary data will also be available retrospectively for the period 0–12 months prior to CCU admission from routine administrative data sets.

**Table 2:** Routine clinical assessment battery and administrative data collection over the baseline, admission, discharge and follow-up collection points

Domain		Measure	Baseline (−12–0 months)	Admission (0–6 weeks post)	Discharge (0–6 weeks prior)	Follow-up (0–12 months)
Administrative data		Health of the Nation Outcome Scale (HoNOS) [26]^A^	X	X	X	X^1^
		Life Skills Profile (LSP-16) [48]^B^	X^*^	X	X	X^2^
		Mental Health Inventory (MHI-38) [51]^B^	X	X	X	X^2^
		Mental health related Emergency Department presentations	X	X	X	X^2^
		Psychiatric inpatient bed days	X^*^	X	X	X^2^
Clinical assessment battery	Symptom measures	Alcohol Use Disorders Identification Test (AUDIT) [60]		X^*^	X	
		Brief Psychiatric Rating Scale (BPRS) [46]		X	X^2^	
		Scale for the Assessment of Negative Symptoms (SANS) [87]		X	X^2^	
	Functional Cognition	Allen’s Cognitive Levels (ACL) [59]		X^*^	X	
	Social-function	Social Functioning Scale (SFS) [88]		X^*^	X^2^	
	Functional	Perceive Recall Plan & Perform System of Task Analysis (PRPP) [89]		X	X	
	Recovery	Stages of Recovery Instrument (STORI-30) [49]		X^*^	X^2^	
	Carer burden	Burden Assessment Scale (BAS) [90]		X	X	
		Adult Carer Quality of Life (AC-QoL) [50]		X	X^2^	

^A^ HoNOS is routinely collected on admission and discharge from inpatient, community residential and ambulatory services, and 3-monthly review periods in the non-inpatient settings

^B^ LSP-16 and MHI are routinely collected on admission, discharge and 3-monthly review in community residential and ambulatory services

^1^ Primary outcome measure, examining change in total score between the Baseline and Follow-up periods

^2^ Planned secondary outcome, examining change between Admission and Discharge for Clinical assessment battery items, and Baseline and Follow-up for Administrative data items

^*^ Planned predictor in hierarchical linear regression modelling, note that for STORI-30 change between Admission and Discharge stage will be used (reduced, stable, increased)

X Collection occasion

### Sample

The project is currently in progress. The cohort will comprise all consumers admitted to the three MSAMHS CCUs (Bayside, Coorparoo and Logan) between December 2014 and December 2017 who provide informed consent to participate. Baseline and follow-up data will be sourced from administrative datasets for the 12-month period prior to and following CCU admission and discharge respectively.

As the study takes a naturalistic observational design, the only defined inclusion criteria is acceptance for a trial of CCU based rehabilitation care. Each CCU provides a six-week assessment period to establish suitability for engagement under the model of service. Given this, consumers who are discharged prior to the conclusion of this assessment period will be excluded from the study. This exclusion criterion assists in ensuring that included participants are aligned with the model of service. This reflects a modified intention-to-treat approach [34], as participants will be included regardless of their mode of exit from the CCU (planned/unplanned (CCU initiated)/unplanned (consumer initiated).

Based on anticipated service throughput, with a median length of stay at the Coorparoo CCU of 0.9 years (inter-quartile range 0.50-1.8 years) at a point estimate in 2013, and assuming a 70 % response rate, it was expected that at least 60 consumers from the integrated staffing model and 40 from the clinical staffing model would agree to participate over the study period. This minimum sample likely reflects an under-estimate given 37 unique admissions occurred to the Coorparoo CCU in the 12-month period from July 2012. Additionally, since the opening of the Bayside and Logan CCUs 50 of the 63 consumers admitted across all three sites met the inclusion criteria (79.4 %) and 42 (84.0 %) of eligible consumers provided consent to participate.

The adequacy of the estimated minimum sample size was considered using the Epi Info™ StatCalc program [35] for unmatched or cross-sectional cohort studies. This minimum sample was estimated to provide greater than 80 % power to detect a 15 % difference in outcome on the primary outcome measure at the 95 % confidence interval using the Fleiss calculation. The acceptability of this power is supported by an emergent difference of 20 % in outcome HoNOS scores in a recent Australian study examining outcomes of a community residential service for similar consumers [36].

### Measures

#### Primary outcome measure

The primary outcome measure will be change in psychosocial functioning as measured by total score on the HoNOS [37]. It is hypothesised that there will be no significant difference in the mean change in highest total HoNOS score recorded in the 0–12 months prior to CCU commencement and the highest score recorded 0–12 months post CCU discharge for consumers under the clinical and integrated staffing model. The total HoNOS score has been selected as the primary outcome measure as it:

1. Has high validity, reliability and sensitivity to change, as well as routinely informing service policy and planning decisions in Australia [38];
2. Is considered appropriate for tracking changes in social functioning over time, and more sensitive in doing so than the CANSAS (a needs based measure) [39];
3. Has an embedded role in individual outcome assessment in Australia and New Zealand [38, 40, 41] and there is evidence that it is being regularly utilised in care planning [42, 43]
4. Is supported by an established national assessment protocol and training processes to facilitate inter-rater reliability [38], and with evidence to support higher levels of acceptability [44] and completion than the other core measures of routine outcome suite [38]
5. It is used and available across multiple international settings [45], increasing the replicability of the methodology.

Utilising the criterion of the highest (i.e. greatest dysfunction) score in the 12-month period pre and post CCU entry limits the risk of conflating the benefits of active rehabilitation support with the benefits associated with support and accommodation in the CCU setting.

### Planned secondary outcome measures and descriptive data

The complexity of outcome assessment in mental health service evaluation and the appropriateness of relying on multiple measures to capture the breadth of relevant domains is well recognised [45]. A routine assessment battery (see Tables 2 & 3) was developed, piloted and adapted at the Coorparoo CCU site in consultation with senior medical and allied health workers over the initial two years of operation (2012–2014). Considerations in the development of this battery included ease of administration, incorporation of multiple perspectives (staff, consumers and carers) and sufficient breadth to capture data on functional, social, cognitive, recovery and symptom related domains.

**Table 3:** Completion of relevant measures and explanation of routine clinical assessment battery

		Responsible person for ensuring completion					
Domain	Measure	Occupational therapist	Principle service provider	Psychology`	Medical	Social work	Notes on assessment battery
Routine outcomes	Life Skills Profile (LSP-16)^a^		X				•These measures form part of the routine assessment battery to be completed with all consumers residing at the Community Care Units. •The battery was developed on the basis of the clinical experience and knowledge at the Coorparoo CCU, with extensive staff consultation. •Standardisation of the assessment process provides an opportunity to better evaluate and compare consumer outcomes, both at the individual and group level. •Whilst there many other relevant measures, the battery chosen aims to broadly cover relevant domains in a manner that does not place excessive demands on consumers, carers and staff. •There will not be an opportunity to change the components of the battery for the duration of the CCU evaluation project. For this reason, it will be critical to find ways in which the chosen measures can be utilised to enhance the quality and experience of care at the units. •It is expected that the measures completed by carers and consumers will form the basis of a subsequent discussion with members of the clinical team. •It is likely that multiple team members may be present at the time a given measure is collected given the multi-disciplinary nature of the work undertaken at the CCUs. •The Principle Service Provider role under both the integrated and clinical staffing models is performed only by clinical staff members. •For the purposes of this study a Carer is considered to be a person (e.g. family member or friend) who the Consumer nominates as having a significant unpaid caring role for them.
	Mental Health Inventory (MHI)^b^		X				
	Health of the Nation Outcome Scale (HoNOS)^a^		X				
Symptom measures	Alcohol Use Disorders Identification Test (AUDIT)^c^				X		
	Brief Psychiatric Rating Scale (BPRS)^d^			X			
	Scale for the Assessment of Negative Symptoms (SANS)^d^				X		
Cognitive	Allen’s Cognitive Levels (ACL)^d^	X					
Social	Social Functioning Scale (SFS)^c^	X					
Functional	Perceive Recall Plan & Perform System of Task Analysis (PRPP)^d^	X					
Recovery	Stages of Recovery Instrument (STORI-30)^b^		X				
Carer burden	Burden Assessment Scale (BAS)^b^					X	
	Adult Carer Quality of Life (AC-QoL)^b^					X	

^a^ Clinician rated measure

^b^ Consumer / carer self-rated measures

^c^ Consumer / carer self-rated measures with the option of oral administration by a staff member

^d^ Clinician rated measure based on interview / assessment with consumer

X Collection occasion

Planned secondary outcome measures will be derived from this assessment battery and routine administrative data sets. Secondary outcome assessment will specifically focus on mean changes in symptomatology (Brief Psychiatric Rating Scale (BPRS) [46] & Scale for the Assessment of Negative Symptoms (SANS) [47]), disability (Life Skills Profile-16 [48]), recovery orientation (Stages of Recovery Instrument (STORI-30) [49]), carer quality of life (Adult Carer Quality of Life (AC-QoL) [50]), emergency department presentations, psychiatric inpatient bed days, and psychological distress and wellbeing (MHI-38) [51]). For each of these secondary measures it is again hypothesised that no differences in mean change will emerge between the clinical and integrated staffing models.

Descriptive details of consumer characteristics and care experience will also be recorded. Relevant information will include: age at CCU commencement, gender, ethnicity, marital status, years of education, diagnosis, involuntary treatment order status, medication, length of stay, and the mode of exit from the CCU (planned/unplanned (consumer initiated)/unplanned CCU initiated).

Information about the operational costs under the integrated and clinical staffing models over the December 2014 - December 2017 period will be reported descriptively. Additional information about the operational experience under the integrated and clinical staffing models including staff retention and absences will also be reported. Estimates of the cost of ongoing service utilisation following CCU engagement based on ED Presentations and acute inpatient bed days in the 12-months post-CCU discharge will be calculated using the most recently available state-wide cost estimates.

### Planned predictors for hierarchical linear regression modelling

A limited number of predictors of positive psychiatric rehabilitation outcome have been supported by the international literature over the past decade. These include: medication adherence [52, 53]; lower baseline disability scores [52]; family engagement with the program and provision of peer support/self-help [52]; increasing age [53, 54]; female sex [54]; being diagnosed with schizoaffective disorder rather than schizophrenia [55] or any disorder other than schizophrenia [52]; and having been engaged with formal education [52]. The contribution of two potentially relevant predictors, namely cognitive function and rehabilitation engagement had not been explored. Cognitive functioning has emerged as a strong predictor in the vocational rehabilitation literature [56] as well as of functional outcomes in schizophrenia generally [57]. There has also been discussion about the importance of considering consumer engagement as a factor in rehabilitation outcomes [16, 17], and there is clear face validity of this concept considering the proportion of consumers receiving support from rehabilitation services who are not engaged with the available support [17, 58] Additionally, alcohol use has historically been the most common non-tobacco related substance use issue arising in Queensland CCUs and has been anecdotally perceived by staff as detrimental to the rehabilitation process.

A total of 15 potential predictors across three logically determined levels (Level 1 – Consumer/Level 2 – Site/Level 3 – Organisational) will be considered (see Table 4). All predictors identified from the recent literature, with the exception of family involvement, are available. Additional plausible predictors to be considered are ‘rehabilitation engagement’ as assessed through the proxy measure of mode of discharge, functional cognition (via Allen’s Cognitive Levels (ACL) [59]), and substance use (via Alcohol Use Disorders Identification Test (AUDIT) [60]). It is hypothesised that consumers who leave the CCU in a planned manner will be more likely to show improvement than those who do not, and that consumers who experience a positive shift in their stage of recovery will be more likely to show improvement. Additionally, it is expected that lower ratings of alcohol related problems at CCU entry will predict favourable outcomes through rehabilitation engagement. Three additional variables relating to the organisation and site (Staffing model/Site/Length of stay) are planned to be included to exclude moderating effects on the Level 1 predictors. Not all of the available predictors will be used in the final multivariate model, as inclusion of such a large number of variables would risk over-fitting to the relatively small sample size [61]. The stopping rule for inclusion of individual predictors will be defined at the significance level of .05.

**Table 4:** Planned variables for inclusion in hierarchical linear regression modelling

Level	Rationale	Construct	Measure/variable name	Type
3 - Organisational	Features with potential impact on consumer experience	Peer support	Staffing model	Dichotomous (Clinical/Integrated)
2 - Site		Site/location	CCU Site	Categorical (Bayside/Coorparoo/Logan)
		Length of Stay	LOS	Continuous
1 - Consumer	Based on previous prediction studies	Demographics	Age	Continuous
			Sex	Dichotomous (Female/Male)
			Education (years)	Continuous
		Primary Diagnosis	Diagnosis	Categorical (Schizophrenia/Schizoaffective Disorder/Other)
		Cognition (functional)	ACL	Scaled
		Medication adherence	LSP-16 (Item 5)	Scaled
		Service utilisation	Psychiatric inpatient bed days	Continuous
		Social function	SFS	Scaled
		Baseline disability	LSP-16 (total)	Scaled
	Novel predictors	Recovery orientation	STORI-30	Ordinal
		Rehabilitation engagement	Mode of discharge	Categorical (Planned/Unplanned - Consumer initiated/Unplanned - CCU initiated)
		Substance use	AUDIT	Ordinal (0-7/8-15/16-19/20+)

### Procedures

Identical processes for data collection will operate at the three sites. The clinical assessment incorporates a combination of clinician, consumer, clinician-and-consumer and carer/family completed measures (see Table 2). For the purposes of this study a Carer is considered to be a person (e.g. family member or friend) who the Consumer nominates as having a significant unpaid caring role for them. Staff for which each measure reflects their core-skills set and routine practice will be responsible for measure completion (see Table 3).

The research protocol was explained to staff prior to the commencement of data collection. Each discipline completing relevant assessments will work to maximise fidelity through regular peer based supervision. Standardised training in the use of routine outcome measures is provided to all staff, this includes opportunities to calibrate scoring of HoNOS and Life Skills Profile-16 (LSP) [48] across the study sites. Fidelity of data relating to symptom measures will be enhanced through the use of the anchored scoring version of the Brief Psychiatric Rating Scale (BPRS-A) [62] and a semi-structured interview guide developed in an Australian context for raters with low-clinical experience [63], as well as a structured interview guide for the Scale for the Assessment of Negative Symptoms (SANS) measure [64]. Inter-rater reliability data will also be calculated from calibration sessions for the BPRS and SANS involving relevant staff.

A research assistant will collate the data from the pre-admission and post-discharge phases from established administrative data sets, where the relevant information is routinely collected.

As is commonly expected in health research [65, 66], it is anticipated that missing data will be present in both the administrative datasets and assessment battery. Categorical codes will be available for non-completed consumer and carer measures, and for clinician rated measures dependent on structured assessment. These codes will designate the reason for non-completion as being due to refusal by the consumer/carer or due to the measure not being offered to the consumer/carer. Missing Values Analysis will be completed in SPSS Version 22 and it is planned for missing data to be handled using the multiple imputation method [67] assuming this is missing at random. Characteristics and patterns of missing data, and the assumed mechanisms will be reported.

### Analysis

The quantitative analysis will be undertaken in SPSS Version 22 and statistical significance will be assessed at a level of 0.05:

1. The cohort of consumers utilising the services across the three sites will be defined using descriptive statistics. Differences between the sites and staffing models will be examined using t-tests and chi-squared analyses. Assuming comparability of the cohorts is established it is planned that the data from the two integrated staffing model sites will be able to be combined.
2. In order to answer the question of equivalence of the integrated and clinical staffing model for CCUs, differences in the total HoNOS score pre admission and post discharge will be assessed using a t-test or Mann–Whitney U test depending on the normality of the distribution.
3. The question of whether CCU rehabilitation care results in change in specific clinically relevant outcome domains, will be answered through analysis of the secondary outcome measures. It is anticipated that data from the secondary outcome measures will be dichotomised and subjected to Mann–Whitney U analysis with appropriate correction for multiple comparisons.
4. Predictors of change in psychosocial functioning following CCU care will be explored using hierarchical linear regression modelling. The dependent variable is the same as the primary outcome variable for the overall study, mean change in total HoNOS score pre admission and post discharge from CCU care. The independent variables will be entered cumulatively with the organisational (Level 3) variable considered first, followed by the site related (Level 2) variables and then the consumer (Level 1) variables. A hierarchical approach is appropriate given the expectation of inter-correlation between the predictors [68].

### Qualitative methodology

#### Design

A pragmatic approach to grounded theory facilitates the dual goals of developing a rich understanding of phenomena under examination as well as allowing for adaptation of service delivery in response to consumer and staff views. The methodology of the qualitative component of the study has been developed with consideration of the COREQ checklist [69], and it is intended that this will also inform the reporting of results. Semi-structured qualitative interviews [70] will engage participants, over time, to gather information on expectations (0–6 weeks post commencement), experiences (12–18 months post-commencement) and reflections (12–18 months post discharge) on care at the site of interest. A matrix detailing the cells for sequential qualitative data collection based on time of collection, CCU site and stakeholder group is presented in Table 5. The opportunities for data collection are partly constrained by the timeframes of service commencement; cells coloured black reflect information not available or not relevant to the planned study. At the time of publication all Phase 1 consumer and staff interviews have already been completed.

**Table 5:** Planned data collection phases, timeframes, focuses and estimated sample size for each cell of data collection

			Clinical staffing model		Integrated staffing model			
			Site 1 (Coorparoo)		Site 2 (Bayside)		Site 3 (Logan)	
	Interview focus	Timeframe	Staff	Consumers	Staff	Consumers	Staff	Consumers
Phase 1	Expectations of care^a^	Commencement		*n* = 4 (6–10)	*n* = 4 (6–10)	*n* = 4 (6–10)	*n* = 4 (6–10)	*n* = 4 (6–10)
Phase 2	Experience of care	12-18 months post-commencement	*n* = 4 (6–10)	*n* = 4 (6–10)	*n* = 4 (6–10)	*n* = 4 (6–10)	*n* = 4 (6–10)	*n* = 4 (6–10)
Phase 3	Reflections on care and transition	12-18 months post-discharge		*n* = 4 (6–10)		*n* = 4 (6–10)		*n* = 4 (6–10)
Number of estimated total interviews			*n* = (6–10)	*n* = (18–30)	*n* = (12–20)	*n* = (18–30)	*n* = (12–20)	*n* = (18–30)
Estimated total sample pool for each site/group (N)			23	20	25	20	20	16

^a^Data on the expectations of care for staff working at the CCU due to this unit being operational prior to study commencement

Numbers presented in brackets reflect the estimated range of interviews for each cell of data collection

#### Sub-sample

A sub-sample of consumers who consent to have their data included in the quantitative analysis from each site will participate in the sequential semi-structured interviews. Within each cell for qualitative data collection, convenience sampling is employed due to the time constraints associated with the collection of expectation data as well as the limited size of the total sample pool in each case. The aim is to be as inclusive as practicable as well as consistent with a grounded theory approach by sampling to achieve thematic saturation. However, the requirements of exhaustive sampling must be balanced against the relatively small sizes of these groups and the need to avoid the possibility of creating real or perceived pressure on staff and consumers to participate if they do not want to. For the integrated staffing model sites, a balance of peer and clinical worker interviews is planned.

A minimum of four interviews will be completed for each cell of data collection prior to determination of the final sample size. We expect the total size for each cell to be between six and ten, but, consistent with a grounded theory approach, sample size will depend on considerations of when, and whether, thematic saturation is reached, as well as the level of interest from each consumer or staff group at each site and time point.

During Phase One consumers commencing at each of the three CCU sites were approached to be involved in the evaluation project during the initial six weeks of their care. Interview participation was prioritized on the basis of earliest provision of consent, and the availability and willingness to complete the interviews at the proposed time. Effort was made to involve the same consumer participants at each of the two subsequent interviewing phases (Phase 2 and Phase 3), with the option to include new participants in the case of attrition or iterative need. Consumer inclusion in the Phase 2 and 3 interviews will be prioritized on the basis of: [1] participation in Phase 1 interviews; [2] balancing consumers who remain resident at the CCU with those who have left; [3] for consumers no longer resident at the CCU balancing those who had planned and unplanned (consumer and CCU initiated) discharges; and [4] availability at the designated interview time. Consumers will be given the opportunity to participate in feedback and discussion sessions regardless of whether or not they were involved in the interviews.

During Phase One staff commencing at the integrated model sites were approached to be involved in the evaluation project in the initial six weeks of their employment. Phase Two will include both the clinical and integrated staffing model sites. Staff interview participation during Phase 1 was prioritized on the basis of achieving a balance of clinical and peer workers from the integrated staffing model sites. Additional considerations in prioritization of interview allocation were the earliest provision of consent and the participant’s availability and willingness to complete the interviews at the proposed time. For the integrated staffing model participants, effort will be made to involve the same staff in the Phase One and Two interviews, with the option to include new participants in the case of attrition or iterative need. Additional considerations in the prioritization of interview allocation for Phase Two staff interviews will be: [1] balancing clinical and peer staff roles at the integrated staffing model sites and [2] proportionate balancing of staff who have stayed on and those who have left at the integrated staffing model sites.

### Procedures

During each interview a trained independent interviewer will encourage participants to elaborate on relevant themes that emerge, and to give their reasons for their perspective and suggestions for improvements to service delivery. Initially an inductive approach will be taken in the analysis of each data cell. Confirmation or refutation of emergent themes and theory will be guided by an inductive-deductive interplay including involvement of staff and consumer participants in feedback and discussion. Data collection, analysis and theorising will occur in tandem from the outset.

Principles of participatory action research [29] have informed the approach, with efforts made to collaboratively engage staff and consumers in the co-creation of knowledge. Attention will be provided to fostering democratic principles, including individual and group discussion with consumers and staff to encourage and maintain buy in and ownership of the qualitative components of the evaluation. The articulation of dissenting opinions will be valued in building the understanding required for incremental improvement to services. Efforts to offset the inherent power differentials affecting consumers in an institutional setting will be made through the use of an independent interviewer and provision of payment for participation. Explicit statements will be made about the role of this gratuity as a token of acknowledgement of the value of consumer involvement. Additionally, the independence of the interviewer, the availability of choice in interview setting, staff review and approval of transcripts, and the de-identification of transcripts will work towards creating a ‘safe space’ for consumers and staff to disclose their personal opinions. Multiple opportunities for participant involvement and feedback will be made available during the analyses, around emergent concepts and potential action.

All interviews will be completed by EN, a research assistant and doctoral level psychology candidate. EN is not involved in the delivery of clinical assessment or care at the CCU sites. CM, an experienced qualitative researcher, has provided training in qualitative interviewing to EN prior to project commencement; and the research team will provide regular supervision and guidance including early review of initial transcripts. The initial interview schedules (Appendix 1) will evolve iteratively in consultation amongst the researchers following each interview. Both consumer and staff interviews are expected to take between 30-min and 1-h to complete.

Consumer interviews for all Phase 1 interviews were conducted in a private non-clinical area on the grounds of the CCU. The option of completing Phase 2 interviews at a neutral site or by telephone will also be available for consumer participants. The AUD$25 gratuity will be provided to consumer participants following each interview in acknowledgement of their participation. All staff interviews will be completed during their work hours in a private room, and for Phase Two interviews staff will be given the option of completing the interview offsite. All interviews will be audio-recorded, independently transcribed by an external transcription company and then de-identified by EN. For the staff interviews the de-identified transcript will be returned via email to participants for approval prior to them being made available to the research team for analysis. During this approval stage staff will be able to edit their transcript. The de-identification of transcripts, conducting interviews in non-clinical areas and providing interview transcripts to staff to review, are measures intended to contribute to the creation of a safe space for participation.

The decision to provide the option of transcript review to staff participants was made based on concerns about the re-identifiability of staff participants, given the relatively small target population and that the investigation was initiated by senior staff within the mental health service. Whilst the interview procedures were designed to establish a safe space for participation, it is foreseeable that staff may experience anxiety about how their information may be utilized and its implication on their personal employment situation. This poses a possible barrier to participation and/or disclosure. Providing additional review overcomes this barrier by allowing staff participants an opportunity to remove any information if they have such concerns. The number of participants who elect to edit their transcripts and the nature of this editing (e.g. addition, redaction, variation or some combination of these) will be detailed in the results. These risks were considered lower for consumer participants, however, the decision not to provide transcripts to consumer participants for review was made for logistic reasons, and reflects a limitation of the methodology.

Interviews at each time point are open-ended and allow the researchers to assess how priorities and preferences, as well as views, change over time. The semi-structured interviews guide the interviewee through topics in historical order, so that they might recall earlier experiences first and more recent experiences last. Open-ended questions are presented initially, with the interview then tapering to more direct prompts. This process allows participants to discuss matters most important and relevant to them, without prejudging what these may be, while also ensuring maximum consistency and comparability in topics discussed across interviews. Both interviewer and interviewees are encouraged to think of the interview as a conversation.

The interviewer will explore emergent themes as the interviews progress, particularly where these appear to diverge from those previously identified. Where an interviewee provides a lengthy answer to a question, the interviewer will paraphrase what they understood to be the key point being made and ask for clarification and confirmation on this. This is anticipated to be important to being able to represent the views of consumers where formal thought disorder or delusional thinking is present and it will also provide additional means of verifying the interviewer’s interpretations of participants’ views. The interviewer will rephrase and simplify questions that are misinterpreted or not understood. In the case of emergent distress, the interviewer will seek ongoing consent for the interview to continue.

### Analysis

The two principal investigators involved in the qualitative aspects of the study, SP and FD, work within the services in clinical leadership roles. From this ‘insider’ position they are theoretically sensitised and familiar with the relevant literature [71]. Potential conflicts relating to the dual role of clinical-researcher are acknowledged. Other members of the research team are engaged from the ‘outsider’ position, with preconceived ideas based on broader knowledge and experience, but not on the specifics of the organisations being evaluated. The involvement of CM and EN in the planning, implementation and review will help facilitate regular discussion and consideration of investigator positionality, and acknowledgement of preconceived beliefs and values of all investigators, to ensure that such prior knowledge does not unduly distort the iterative analysis of empirical material collected.

De-identified transcripts will be uploaded into an electronic database (NVivo10) [72] for analysis. Multiple researchers (SP, FD, EN, CM) will review the initial transcripts for each data-collection phase, working collaboratively towards consensus on an initial coding scheme. This discussion will inform revision of the interview schedule and final decisions around sample sizes to achieve thematic saturation. The coding process will pay attention to, and distinguish between, first order themes relating to concrete and easily articulated issues and experiences (e.g. specific concerns about infrastructure, work conditions and/or interventions) as well as second order themes that may be more nebulous (e.g. pertaining to identity, aspirations and relationships). The research team will work collaboratively to consider limitations in the coding, and identify instances where theorising ceases to be adequately grounded in the available data. Multiple tactics will be consciously applied to generating meaning through analysis at each cell of the data collection [73]. These tactics will include generating meaning, testing and confirming hypotheses and considering the quality of the conclusions.

In the case of apparent convergence between the two integrated staffing model sites it is anticipated that the data will be analysed in composite for both staff and consumers at these sites. The resultant themes will be used to examine the similarities and differences between staff and consumer expectations and experiences of care under the two staffing models. Additionally, comparison and efforts to link consumer expectations, experience and reflections on care under the two staffing models will be used to develop our understanding of the consumer journey through these units. Feedback on both the content and concepts raised in interviews will be presented at least 6-monthly to consumer and staff groups at each site for feedback and consideration of relevant adaptations to service delivery that can be made. A record will be kept of changes to service delivery based on feedback at each time point in order to provide relevant context and depth to the analysis. Draft manuscripts will be made available to interested participants for comment prior to submission for publication.

### Quantitative-qualitative synthesis

Comparison of emergent themes relating to the integrated and clinical staffing models will inform the understanding and interpretation of the quantitative findings. Matching of participant identification codes for quantitative and qualitative data will allow focused exploration of experiential aspects of groups of consumers identified as benefiting and non-benefiting from CCU support. Additionally, the Phase 2 and 3 interview schedules may be adapted in response to initial data and analyses from the quantitative data-set.

## Discussion

The protocol is novel in both the focus of investigation as well as the longitudinal, multi-perspective approach with intentional combination of quantitative and qualitative consumer data over multiple time points. This approach allows a rich understanding of the dynamic nature of the phenomena under consideration. The naturalistic design increases the relevance of the study outcomes to the delivery of complex interventions to consumers in real world clinical settings. The resultant data set will facilitate understanding of whether equivalent outcomes can be realized under a staffing model where the majority of the members of the rehabilitation team are employed on the basis of lived experience of mental illness, as well as how this staffing model impacts the experience of care.

A conceptual limitation of the study is the emphasis on the CCU and it staff as a key consideration in the outcomes of people with a diagnosis of serious and persistent mental illness. The factors impacting on entry to, exit from and the outcomes of rehabilitation care are likely multifactorial and dependent on resources and supports external to the unit [74]. Considerations in the socio-economic environment include the availability of housing, employment and welfare opportunities; funding of external clinical and non-clinical services, and broader issues relating to social and structural stigma impacting functional outcomes. Exploration of these external factors is beyond the scope of the present study, but may emerge in the qualitative analysis.

Several limitations to the quantitative aspects of the methodology need to be considered. The primary outcome (HoNOS) measures constructs of relevance to clinically defined recovery outcomes rather than personal recovery outcomes or the recovery process. Reliance on the HoNOS in Australia has been criticized with reference to its focus on consumers deficits and problems [38]. Additionally, the lack of correlation between this measure and consumer defined recovery measures has been noted [75]. However, it has been suggested this finding is best explained in relation to the difference between clinical and consumer rated measures [76]. Both the qualitative and secondary quantitative analyses will be important in interpreting the meaningfulness of observed change or failure to observe change in the primary outcome measure.

There are inherent limitations with relying on routinely collected administrative and clinical data, including missing data and recording bias [36]. Furthermore, the absence of blinding of raters provides a potential source of bias, however the primary outcome assessments in during the baseline and discharge periods will be completed by trained assessors independent of the CCU context. The absence of randomized allocation between the staffing models hinders our ability to make causal inferences regarding relationships between service provision and changes in outcome over time. However, the inappropriateness of running randomized placebo-controlled trials in socially complex services with reference to ethical and practical barriers has been acknowledged in the literature [30]. Regardless, the utility of equivalence testing depends in part on the underlying assumption that the comparator has been convincingly proven to have superior efficacy to placebo [77]. This assumption cannot be definitively asserted on the basis of the available literature.

The naturalistic approach to sample selection confers both benefits and costs. Whilst the defined exclusion criterion aims to facilitate inclusion of only participants initially deemed to align with the model of service, this inclusive approach risks masking the effectiveness and differences between the two treatment arms through the inclusion of participants who do not complete their planned rehabilitation. Whilst the option of a per-protocol analysis approach [34], including only those participants whose CCU discharge was planned, will be available such an approach would not reflect the reality of rehabilitation practice. It has been argued that limited benefits are to be expected through provision of rehabilitation care without consumer engagement in the process [78]. There is an emerging interest in engagement scales for use in residential rehabilitation settings, including adaptation of an established measure used in assertive community treatment [16]. However, the broad suitability of this measure in application has not yet been established. Engagement with rehabilitation support over the CCU stay is not being specifically measured in this study. It is anticipated that the qualitative interviews with consumers will provide rich detail about relative levels of engagement under the two staffing models. Length of stay (whether a consumer remains for the minimum 6-month length of stay detailed in the model of service) will provide an additional proxy measure of engagement.

The longitudinal approach to sampling adds additional complexity to the evaluation. There is an expectation that service delivery will change over time. Generalizability of findings may also be affected by the expectation of incomplete sampling and site related differences in referral patterns. Given this, careful consideration of the characteristics of participants and non-participants at each is site is warranted.

The qualitative aspects of the methodology deviate from a purist reading of grounded theory insofar as it acknowledges the applied importance of reporting (largely descriptive) content as part of a formative evaluation. However, the methodology does work towards fulfilling the key criteria by which qualitative research is judged i.e. credibility, originality, resonance and usefulness [79]. The approach provides opportunity to derive both theory and describe content that is relevant and responsive to the needs of mental health service planners, staff and consumers. Practical and theoretical complexities associated with longitudinal qualitative analyses revolve around the task of constructing a consistent narrative that weaves through expectations to reality and reflections on care at the CCUs while preserving the ability to represent changes in participants’ priorities and preferences over time. It cannot be assumed that what is important and relevant to staff and consumers will remain stable, and that the evaluation can simply measure changes in their views on matters raised at the beginning of their journey. The analyses need to be sensitive to the evolving nature of the services under investigation. These changes need to be documented over the course of the study.

The qualitative component of the study does not include the voice of carers. Carers are identified as a key stakeholder in mental health service provision [80]. The omission of carers from the qualitative analysis reflects pragmatic limitations in the ability to realise a comprehensive longitudinal evaluation. Future exploration of the expectations and experiences of CCU based care from the perspectives of carers is encouraged, and it is possible that divergence from the views of both staff and consumers may emerge.

Multiple sources of potential bias need to be considered in the qualitative analysis. Retrospective biases are likely to impact comparison recall of alternative care and work settings for both consumers and staff [81]. There are specific challenges associated with the consumer sample. The risk of deficits and bias in the self-report of persons with schizophrenia, especially around functional capacity, are well documented [82]. There is also a risk that the provision of a gratuity to consumers creates an undue incentive to participate [83], and that a self-selected and financially incentivized consumer group do not adequately reflect the general population of service users [84]. The inherent power differential associated with the consumer role within the service under investigation creates a risk of pseudo-participation [29]. Additionally, for staff participants the dual roles of several members of the research team may inhibit more negative disclosures about their work experiences. Given these issues, careful and ongoing attention to the collaborative involvement of participants and the creation of a safe space for participation is needed.

There is very limited research exploring the outcomes and experience of community based residential mental health rehabilitation care. The generalisability of results from the present study will be limited by the focus on a single model of service and two sites for the integrated staffing and a single site for the traditional clinical staffing model. It is possible that idiosyncratic aspects of practice at each site or under the two staffing models will impact on the outcomes of the study. However, the richness of detail facilitated by taking a longitudinal mixed methods approach incorporating multiple stakeholder perspectives is anticipated to facilitate relevant inferences to guide practice in alternative contexts.

## Abbreviations

ACL, allen’s cognitive levels; AC-QoL, adult carer quality of life; AUDIT, alcohol use disorders identification test; BPRS, brief psychiatric rating scale; BPRS-A, brief psychiatric rating scale – annotated; CCU, community care unit; LSP, life skills profile - 16; MHI-38, mental health inventory - 38; MSAMHS, metro south addiction and mental health service; NFROMHS, national framework for recovery oriented mental health service; NGO, non-government organisation; PSW, peer support worker; RANZCP, royal Australian and New Zealand college of psychiatrists; SANS, scale for the assessment of negative symptoms; STORI-30, stages of recovery instrument.

## Appendix 1

### Evaluating residential mental health rehabilitation outcomes across the community care units

#### Initial interview schedule (for semi-structured individual interviews)

Note that the listed questions and associated prompts are intended to guide the initial interviews at the Community Care Unit (CCU) for both staff and consumers. These questions will be utilised by the interviewer for at least the first 4–6 interviews for consumers and staff in both the clinical and integrated staffing settings. The interview schedule will be adjusted on the basis of emergent themes and the adequacy of data following this time. It is expected that the listed questions and associated prompts for Phase 2 and 3 data collection will be significantly adapted based on emergent themes and hypotheses over the progression of the project.

Additional guidance:

1. Explore emergent themes as the interview progresses, particularly where these appear to diverge from those previously identified.
2. Where an interviewee provides a lengthy answer to a question, paraphrase what you understand to be the key point being made and ask for clarification and confirmation on this.
3. In the case of emergent formal thought disorder or delusional thinking rephrase and simplify questions that appear to have been misinterpreted or not understood.
4. In the case of emergent distress seek ongoing consent for the interview to continue, at the conclusion of the interview offer additional support as outlined in the Consumer and Staff Information and Consent Forms.

### PHASE 1

#### Questions (and associated prompts) for initial interviews with staff

Thank-you for agreeing to be interviewed. Before we get started I’d like to invite you to think about the interview as a conversation.

While we have a set of questions we’d like to ask you, we are interested in hearing your perspectives on the topics we raise in your own words. The questions we ask have no right or wrong answers and we invite you to provide us with as much, or as little, detail as you would like.

We are interested in how you have come to be here at the Community Care Unit and what you are expecting it to be like.

1. How do you think this experience will compare to previous mental health settings where you have worked?
  - In what settings have you worked before?
  - What have you heard about working here?
  - Do you think working at CCU will be different to your previous experiences? In what ways/why not? Do you think it will be better, worse or the same as your previous experiences? Why/why not?
  - Do you expect that the CCU as a place/staff/consumers/processes will be the same or different? In what ways/why not?
2. What are your expectations of the Community Care Unit (CCU) experience?*
  - In your own words, can you tell me what you think the CCU is?
  - What do you expect the CCU will be like?
  - What do you imagine it will be like for resident to be here?
  - Are you looking forward to working here? What are you looking forward to? Why?
  - Is there anything you not looking forward to about here? What sorts of things? Why?
  - Do you have any concerns about working here? What are these? Why? (In the case of reference to conflict, explore values) What kind of conflicts do you think might arise here?
  - What do you hope to achieve here? (If discussing change for consumers) Why is achieving this important to you?
3. Why have you chosen to work here?
  - What other options for working within the mental health sector or with people experiencing severe and persisting mental illness are you aware of?
  - What other options were available to you?
  - Why did you choose the CCU option over the others you were aware of?
  - Is this an attractive place to work, and if so why?
  - What are you hoping the CCU will be like?

* If they refer to ‘recovery’, then explore further, e.g. What does recovery mean to you/The concept of recovery can mean many different things to different people, what does it mean to you?

### PHASE 1

#### Questions (and associated prompts) for initial interviews with consumers

Thank-you for agreeing to be interviewed. Before we get started I’d like to invite you to think about the interview as a conversation.

While we have a set of questions we’d like to ask you, we are interested in hearing your perspectives on the topics we raise in your own words. The questions we ask have no right or wrong answers and we invite you to provide us with as much, or as little, detail as you would like.

We are interested in how you have come to be here at the Community Care Unit and what you are expecting it to be like.

1. How do you think this experience will compare to your previous experiences of mental health care?
  - In what kinds of places have you received mental health care before?
  - What have you heard about living here?
  - Do you think living here will be different to places you have been before? What might be better/worse?
  - Do you expect that the place/staff/daily life will be the same or different?
2. What are your expectations of the Community Care Unit (CCU) experience?*
  - In your own words, can you tell me what you think the CCU is?
  - What do you expect the CCU will be like?
  - Are you looking forward to living here? What are you looking forward to? Why?
  - Is there anything you like about the CCU?
  - Is there anything you not looking forward to about living here? What sorts of things? Why?
  - Is there anything that you don’t like about the CCU?
  - Do you have any concerns about living here? What are these? Why?
  - Is there anything that you would change about the CCU?
  - What do you hope to achieve here?
3. How did you come to be here at the CCU?
  - What other places do you know where you could have received mental health support/you could be living?
  - What other options were made available to you?
  - Was it your decision to come to the CCU? If not, who made the decision? If yes, why did you choose the CCU option over the others you were aware of?
  - Is this an attractive place to live, and if so why?
  - What are you hoping the CCU will be like?

* If they refer to ‘recovery’, then explore further, e.g. What does recovery mean to you/The concept of recovery can mean many different things to different people, what does it mean to you?

### PHASE 2

#### Questions (and associated prompts) for initial interviews with established staff

Thank-you for agreeing to be interviewed. Before we get started I’d like to invite you to think about the interview as a conversation.

While I have a set of questions we’d like to ask you, I am interested in hearing your perspectives on the topics raised in your own words. The questions have no right or wrong answers, please provide us with as much, or as little, detail as you would like.

We are interested to know your thoughts and experiences of the Community Care Unit, as well as any thoughts or suggestions that might make the CCU a better place to work and to support residents.

1. What do you think the CCU is all about?*
  - In your own words, can you tell me what you think the CCU is?
  - What is it like to work here?
  - What do you imagine it is like for residents to be here?
  - Do you look forward to coming to working here each day? Why?
  - Are there any things that you don’t looking forward to about coming to work here? What sorts of things? Why?
  - Do you have any concerns about working here? What are these? Why?
  - What do you hope to achieve here?
2. How does/did the reality of working here compare to what you had imagined when you had started?^
  - What has met your expectations?
  - What has not met your expectations?
3. How does/did this experience compare to previous mental health settings where you have worked?
  - In what settings have you worked before?
  - What do others think about you working here?
  - Is working at the CCU different to your previous experiences? In what ways/why not? Is it better, worse or the same as your previous experiences? Why/why not?
    Is the CCU as a place/staff/residents/processes the same or different? In what ways/why not?
4. Why do you continue to work here/Why did you stop working here?
  - What other options for working within the mental health sector or with people experiencing severe and persisting mental illness are you aware of?
  - What other options are available to you?
  - Is the CCU preferable to the other options you are aware of?
  - Is this an attractive place to work, and if so why?
  - Have you thought about leaving, if so why?
  - What changes might make the CCU a better place to work/for consumers/for carers (if not previously addressed)?

* If they refer to ‘recovery’, then explore further, e.g. What does recovery mean to you/The concept of recovery can mean many different things to different people, what does it mean to you?

^ If at the conclusion of the interview the staff member has not made reference to key points raised in the Phase 1 integrated staff interviews relating to expectations of working at the CCU a prompt will be provided in relation to these.

### PHASE 2

#### Questions (and associated prompts) for initial interviews with established consumers

Thank-you for agreeing to be interviewed. Before we get started I’d like to invite you to think about the interview as a conversation.

While I have a set of questions we’d like to ask you, I am interested in hearing your perspectives on the topics raised in your own words. The questions have no right or wrong answers, please provide us with as much, or as little, detail as you would like.

We are interested to know your thoughts and experiences of the Community Care Unit^#^, as well as any thoughts or suggestions that might make the CCU a better place to receive rehabilitation support.

1. How does/did this experience compare to previous mental health settings where you have received care?^
  - In what settings have you received care before?
  - What do others think about you being here?
  - Is being at the CCU different to your previous experiences? In what ways/why not? Is it better, worse or the same as your previous experiences? Why/why not?
  - Is the CCU as a place/staff/residents/processes the same or different? In what ways/why not? If peer support workers are explicitly mentioned explore how this has affected the CCU experience.
2. What do/did you think the CCU is all about?*
  - In your own words, can you tell me what you think the CCU is?
  - What is it like to live here?
  - What do you imagine it is like for other residents to live here?
  - What do you imagine it is like for staff to work here?
  - Do you enjoy being here each day? Why?
  - Are there any things that you don’t enjoy about being here? What sorts of things? Why?
  - Do you have any concerns about living here? What are these? Why?
  - What have you achieved here? What do you hope to achieve?
3. How does/did the reality of being here compare to what you had imagined when you had started?^
  - What has met your expectations?
  - What has not met your expectations?
4. Why do you continue to live/receive rehabilitation support here? OR How did you come to leave here?
  - What other rehabilitation/support/accommodation options are you aware of?
  - What other options are available to you?
  - Is the CCU preferable to the other options you are aware of?
  - Is this an attractive place to live, and if so why?
  - Have you had thoughts about leaving, if so why/where/when?
  What changes might make the CCU a better place to live/receive rehabilitation support/for staff/for carers (if not previously addressed)?
5. What are/were your thoughts about leaving here?
  - Where would you like to live in the future?
  - Will it be easy or difficult to leave the CCU?
  - What help do you think you will need when you leave the CCU?

^#^ If the participant is no longer a resident at the CCU the interviewer may need to redirect them to discussion of their experience of being at the CCU. The interview should focus on their reality of being at the CCU rather than exploration of the post-CCU experience (which occurs in Phase 3).

* If they refer to ‘recovery’, then explore further, e.g. What does recovery mean to you/The concept of recovery can mean many different things to different people, what does it mean to you?

^ For integrated consumers only - if at the conclusion of the interview the consumer has not made reference to key points raised in the Phase 1 integrated consumer interviews relating CCU expectations a prompt will be provided in relation to these.

### PHASE 3

#### Questions (and associated prompts) for initial interviews with discharged consumers

Thank-you for agreeing to be interviewed. Before we get started I’d like to invite you to think about the interview as a conversation.

While I have a set of questions we’d like to ask you, I am interested in hearing your perspectives on the topics raised in your own words. The questions have no right or wrong answers, please provide us with as much, or as little, detail as you would like.

We are interested in your reflections on the experience the Community Care Unit, and how you have gone since you left the unit. We are also interested in any thoughts or suggestions that might make the CCU a better place for others to receive rehabilitation support in the future.

1. How have things been for you since you left the CCU?*
  - How do you spend your time (family/friends/work/volunteering/clinical contact)? Are you satisfied?
  - Tell me about where you have been living, what is it like? How well are you managing? Would you like to be living somewhere else?
  - What support are you getting? What support do you think you need?
  - How is your mental health care similar or different now to when you were at the CCU?
2. How well did the CCU prepare you for living in the community?
  - Are things better/worse/the same as they were before you went to the CCU?
  - Were there any important things that happened at the CCU (co-residents/staff/interventions/events)? What made this important? If peer-support workers are specifically mentioned explore how this impacted (positively/negatively) on the experience.
  - What do you think helped? What do you think did not help?
  - Have your expectations/goals been met. If yes – explore how these have been met. If no – explore why not.
3. How could your experience at the CCU have been improved?
  - What was done well at the CCU? What was not done well at the CCU?
  - Would you recommend the CCU to a friend or family member? Is so, why? If not, why not?

* If they refer to ‘recovery’, then explore further, e.g. What does recovery mean to you/The concept of recovery can mean many different things to different people, what does it mean to you?
